# Multiple Targets Directed Multiple Ligands: An In Silico and In Vitro Approach to Evaluating the Effect of Triphala on Angiogenesis

**DOI:** 10.3390/biom10020177

**Published:** 2020-01-23

**Authors:** Chandran S. Abhinand, Prabhakaran A. Athira, Sasikumar J. Soumya, Perumana R. Sudhakaran

**Affiliations:** 1 Department of Computational Biology and Bioinformatics, University of Kerala, Thiruvananthapuram, Kerala-695581, India; abhinand.rohini@gmail.com; 2 Inter-University Centre for Genomics and Gene Technology, University of Kerala, Thiruvananthapuram, Kerala-695581, India; athiraprabha@gmail.com (P.A.A.); soumisj@gmail.com (S.J.S.)

**Keywords:** angiogenesis, triphala churna, polyherbal formulation, molecular docking

## Abstract

Angiogenesis is critical in both physiological and pathological conditions and targeting angiogenesis is a promising strategy for the development of therapies against cancer; however, cells develop resistance to anti-angiogenic therapy, necessitating a more effective strategy. Natural medicines have been used in anti-cancer therapy for many years, but the mechanisms behind these have not generally been explored. Triphala churna (THL), an Indian ayurvedic herbal formulation made from the dried fruits of three medicinal plants, is used as a herbal drug for the treatment of various diseases, including cancer. THL contains over fifteen phytochemicals with different pharmacological effects, especially inhibition of tumor progression. In this study, we examined the effect of these compounds against different targets using docking and in vitro studies. Results showed that THL has a prediction efficacy of (−)436.7, and it inhibited angiogenesis by blocking multiple components of the VEGF/VEGFR2 signaling pathway. The anti-angiogenic effect was mediated by the combined effect of the two top ranked phytochemicals, punicalagin (−424.8) and chebulagic acid (−414.8). The new approach developed in this study to determine the potential efficacy of herbal formulation could be a useful strategy to assess the efficacy of different herbal formulations.

## 1. Introduction

Angiogenesis, the formation of new blood vessels from existing vasculature, is an important process required during post-natal life and is regulated by pro- and anti-angiogenic factors [1]. Shifts in the balance between these factors can lead to excessive or insufficient angiogenesis, contributing to the pathogenesis of cancer, cardiovascular diseases, and other inflammatory diseases. Angiogenesis is critical for tumor progression as it provides nutrients that feed disease tissue [2]. Tumor growth and angiogenesis are codependent; tumor cells secrete several pro-angiogenic molecules that signal endothelial cells to trigger neovascularisation. Development of hypoxia in growing tumor tissue acts as a stimulant for the predominant growth of malignant tumor by inducing the production of pro-angiogenic regulators [3]. Vascular endothelial growth factor (VEGF) is an important endothelial-specific pro-angiogenic growth factor and also a vital mediator of tumor angiogenesis, the expression of which is subject to regulation by multiple factors, including the status of oxygen and metabolites, and is altered under tumor conditions [4]. Carcinomas are characterized by increased expression of VEGF and its receptors, which facilitates metastasis [5]. VEGF increases microvascular permeability, inducing endothelial cell proliferation, migration, survival, and secretion of matrix degrading enzymes [6]. The role of VEGF in tumor angiogenesis and its neutralization by VEGF antibodies have been reported previously; dominant negative VEGF receptors inhibit both angiogenesis and progression of the tumor [7]. Anti-angiogenic drugs, in particular anti-VEGF therapies, have entered the clinical armory against cancer. Different pharmaceutical compounds that target vascular endothelial growth factor receptor (VEGFR) signal transduction are available. Bevacizumab/Avastin is one of the most commonly used angiogenic inhibitors [8]. However, resistance to anti-VEGF therapy is developed by certain tumors, which leads to the development of capillaries at the tumor site, favoring its growth and proliferation. Cells develop resistance to anti-VEGF therapy either due to alternate mechanisms which lead to VEGF activation, or due to signaling in a VEGF-independent manner. Synchronous activation of various signaling pathways involving a number of signaling molecules and the interaction between signaling networks contribute to the modulation of angiogenesis. We developed a pathway map of VEGF/VEGFR2 signaling relevant to angiogenesis [9]. It consists of critical junctions where various downstream signaling pathways converge and communicate by regulatory feedback and feedforward loops involved in the modulation of angiogenesis. Moreover, there is crosstalk of VEGF signaling with other extrinsic signaling pathways which interact synergistically or antagonistically. It therefore appears that a single drug that targets a single site may not be that effective.

Natural bioactive compounds are gaining much attention in therapeutics for their ability to regulate various biological processes that underlie several disease conditions. Angiogenesis being a potential target in the treatment of a number of pathological states, the search for compounds from natural sources that can affect angiogenesis is of great interest. Reports have shown that many naturally occurring phytocompounds exhibit anti-angiogenic activity [10]. Triphala churna (THL) is a polyherbal formulation containing multiple pharmacologically active components and it is used in traditional systems of medicine for the treatment of various diseases [11,12,13]. It is made up of dried fruits of three medicinal plants, *Terminalia bellirica*, *Terminalia chebula*, and *Embelica officinalis*, in equal proportions. Recent studies have shown that THL has anti-tumor properties in mice [14]. However, the mechanisms by which THL mediates its anti-tumor actions are still being explored. Earlier in vitro studies using extracts of THL [10], and a study from our laboratory on chebulagic acid (CA), one of the important components of THL, showed an anti-angiogenic effect [15]. CA, a COX/LOX dual inhibitor, is reported to inhibit angiogenesis by modulating the VEGFR2-dependent GSK-3β signaling pathway [16,17]. Triphala contains over fifteen phytochemicals with different pharmacological effects [18], but the effects of individual phytochemicals are not yet known. Although in silico docking studies with single targets and multiple ligands have been found to be useful for the prediction of the relative efficacies of synthetic compounds, this approach is not adequate to examine the efficacy of polyherbal formulations, which have multiple phytocompounds acting on multiple targets. The effects of the different components of Triphala churna on angiogenesis were examined using various computational tools and validated in cell-based assays, and the results are presented below.

## 2. Materials and Methods

### 2.1. Materials

MCDB 131 medium, antibiotic antimycotic solution, o-phenylenediamine dihydrochloride, Tris base, protease inhibitor cocktail, punicalagin, monoclonal antibodies against CD31, E-selectin, VEGF, FGF, Akt, phosphoserine, and horseradish peroxidase (HRP)-conjugated secondary antibody were purchased from M/s Sigma Aldrich Co. Tissue culture plasticware was procured from M/s. NUNC, Denmark. Nitrocellulose membrane was purchased from M/s. Bio-Rad Laboratories, USA. All other reagents of analytical grade were from M/s. Merck Ltd., Mumbai, India. Triphala churna was purchased from M/s. Dabur, India.

### 2.2. Methods

#### 2.2.1. Docking Studies

##### Target Selection and Preparation

We mainly focused on the VEGF/VEGFR2 pathway, and selected the potential targets based on the pathway information from the published database [9]. X-ray crystallographic structures of various components of the VEGF-dependent angiogenesis pathway were retrieved from protein data bank (PDB). For docking studies, the raw proteins were further prepared by deleting water molecules, ligands, and hetero atoms and adding hydrogen to polar groups using Discovery Studio 4.0 and MGL Tools 1.5.6.

##### Ligand Preparation

The phyto profiles of three herbs in THL (*Terminalia bellirica*, *Terminalia chebula*, and *Embelica officinalis*) were collected from the literature review and fifteen phytochemicals were selected. The chemical structures of these phytochemicals were retrieved from Pubchem compound database and were prepared for the docking studies using MGL Tools 1.5.6, and converted to PDBQT file format for input in to AutoDock vina 1.0.2.

##### Molecular Docking

Molecular docking calculations were done using AutoDock vina, since it permits robust molecular docking with high efficiency and is also an open-source program. Target proteins were docked against phytocompounds using AutoDock vina 1.0.2 to explore the binding affinity and interactions. In general, the docking parameters for AutoDock Vina were kept as the default values. The size of the docking grid was 40 Å × 40 Å × 40 Å along the x, y, and z axes, respectively, and the exhaustiveness was 8. The molecular docking was conducted according to the default protocol and binding affinity was analyzed.

##### Prediction Model

The prediction efficacy (PE) of a phytochemical for angiogenesis was calculated as the sum of its binding affinity values with all the targets obtained by docking with Autodock Vina 1.0.2, using Equation (1). Based on the affinity value, we developed a drug–target network (DTN) connecting phytocompounds and their target proteins, which provided a general idea about the polypharmacology of the compounds [19].

(1) $$\mathrm{PE}\;\mathrm{compound}\;=\;\underset{x\in P}{\sum }\;\mathrm{score}\;x$$

where ‘P’ is the set of proteins related to angiogenesis and ‘x’ the docking score between this compound and the xth protein.

Similarly, the prediction efficacy of the polyherbal formulation was defined as the sum of the PE values of all the compounds contained in this formulation, Equation (2):

(2) $$\mathrm{PE}\;\mathrm{compound}\;=\;\overset{N}{\underset{j}{\sum }}\;\mathrm{score}\;j$$

where N denotes the number of compounds with high docking scores for each target in the signaling pathway.

##### Screening Targets

To examine the mode of action of highly active phytochemical in THL, molecular docking studies were carried out against crucial targets in VEGF-mediated angiogenesis using the LIBDOCK protocol of Discovery studio 4.0. This protocol allowed flexible docking of ligands with receptors and generated different poses for each ligand.

#### 2.2.2. Angiogenesis Assay

Endothelial cells from human umbilical vein (HUVECs) were used as described previously [20]. Endothelial cells were maintained in culture in MCDB medium supplemented with 10% FBS in a Sanyo carbon dioxide incubator at 37 °C, 95% air, 5% CO_2_ atmosphere.

#### 2.2.3. Cell Migration Assay

Cell migration assay was performed according to Liang et al. [21]. Endothelial cells were maintained in culture in endothelial growth medium in a 24 well plate and kept in a cell culture incubator. A scratch was made in the culture dish and the detached cells were washed out. Cells were treated with the sample of interest and cell migration was observed at regular time intervals. Photographs were taken, Image J Macros software was used to measure the width of the wound, and the gap closure was expressed as migration rate in percent.

#### 2.2.4. Enzyme-Linked Immunosorbent Assay (ELISA)

Indirect ELISA was carried out as described by Engvall and Perlman [22]. Proteins were probed with specific primary antibody and HRP-conjugated secondary antibody. Color was visualized using o-phenylenediamine dihydrochloride as chromogen. Protein was estimated using Lowry’s method [23].

#### 2.2.5. Western Blot

Western blot analysis was performed according to Towbin et al. [24]. SDS-PAGE was performed and the proteins were transferred from gel to the NC membrane and probed with specific primary antibody (dilution 1:1000) and secondary antibody (HRP-conjugated) (dilution 1:1000). Diaminobenzidine was used as the chromogen for visualizing bands. The intensity of bands was analyzed using image analysis software (Biorad gel documentation system) and plotted.

#### 2.2.6. Statistical Analysis

Results are expressed as mean with standard error of mean. The statistical significance of difference was analyzed with Student’s *t*-test and one-way ANOVA using GraphPad Prism (Version 5) software. A value of *p* < 0.05 was considered significant.

## 3. Results

### 3.1. In Silico Identification of Drug Targets against VEGF-Mediated Angiogenesis

VEGF signaling is one of the key pathways mediating angiogenesis; we examined the effect of the polyherbal formulation THL on various protein components of this signaling pathway. Docking studies were performed using various phytochemicals of THL on 27 potent targets. The results of the docking of each of the 15 phytochemical against these different protein targets are given in Table 1. While punicalagin showed highest binding affinity to most of the targets (VEGF A, VEGF R1, VEGFR2, NRP 2, PKC γ, MEK, ERK, PLC γ, FAK, Cdc42, RAC, AKT/PKB, eNOS, Hsp27, Axin, GSK 3β, MMP9, MMP3), chebulagic acid showed best binding affinity to seven targets (NRP 1, RAF 1, p38MAPK, SRC, PI3K, paxillin, β-catenin) and isoterchebulin showed the highest binding affinity to two targets (RAS, MAPKAPK) of the 27 selected components of the VEGF/VEGFR2 signaling pathway. Results showed that most of the active compounds present in THL had some binding affinity to each of the target proteins, which implies that the mode of action of THL involves a combined effect of these active components.

These results were further used to calculate the prediction efficacy of individual components of THL. A threshold value was set as (−) 6.0 to filter out the best docked poses. Punicalagin showed the highest efficacy (−424.8), followed by chebulagic acid, isoterchebulin, chebulinic acid, and corilagin in that order, to other components. Reports have shown that the relative levels of these compounds are high in THL compared to other constituents [12]. The prediction efficacy of THL against angiogenic targets, which was calculated by summing up the highest binding affinities of individual targets with 15 phytochemicals, was found to be (−) 436.7. The results are shown in Table 2.

### 3.2. In Silico Identification of Drug Targets against Inflammation

Since angiogenesis and inflammation are codependent, docking studies were done to measure the binding affinities of various phytochemicals of THL on potent mediators of inflammation, namely COX-2 (3LN1) and 5-LOX (3O8Y).

These results were further used to calculate the prediction efficacies of individual components of triphala. A threshold value was set at (−) 6.0 to filter out the best docking poses, and the results are shown in Table 3. Punicalagin showed the highest efficacy (−33.8) to COX-2 and 5-LOX, followed by chebulagic acid and isoterchebulin, in that order, to other components. Further, the prediction efficacy of THL against these inflammation targets which was calculated by summing up the highest binding affinities of individual targets with 15 phytochemicals, was found to be (−) 33.8. The results are shown in Table 4.

### 3.3. Effect of Ethanolic Extract of Triphala Churna on Markers of Angiogenesis

**Figure 1:**
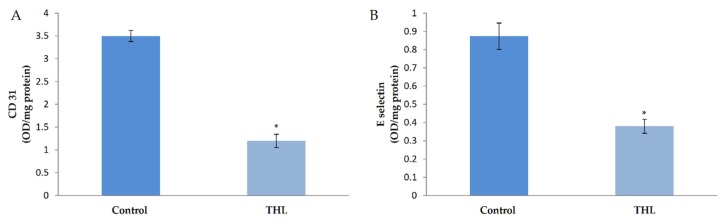
Effect of triphala on the production of CD31 and E-selectin by HUVECs. HUVECs were maintained in culture in MCDB 131 medium supplemented with ethanol extract of triphala (25 µg/mL) for 48 h. The levels of cell-associated CD31 (**A**) and E-selectin (**B**) in the medium were estimated by ELISA using anti-CD-31 and anti-E-selectin respectively. Values given are the average of five experiments ± SEM. * statistically significant compared to control (*p* < 0.05).

In order to examine the effect of triphala churna on angiogenesis, HUVECs in culture were supplemented with ethanolic extract of THL and the production of biochemical markers of angiogenesis, namely CD31 and E-selectin, was analyzed. ELISA showed a significant decrease in the amount of CD31 in cells treated with ethanolic extract of THL in the presence of serum compared to control (Figure 1). Results of the study on the effect of THL on CD31 production by western blot analysis are given as supplementary data, and showed that THL reversed the VEGF-induced upregulation of CD31 (Supplementary Figure S1). The level of E-selectin in the medium was analyzed and the results showed that E-selectin was downregulated in cells treated with THL extract in the presence of serum compared to control. These results are shown in Figure 1.

### 3.4. Effect of Ethanolic Extract of Triphala Churna on the Production of Angiogenic Growth Factors by HUVECs in Culture

**Figure 2:**
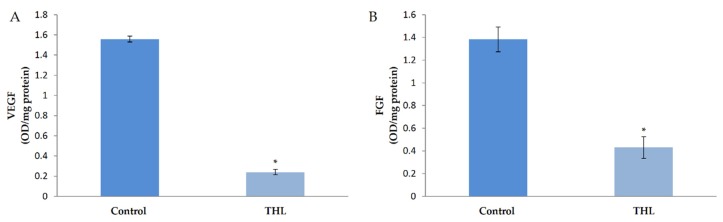
Effect of triphala on the production of VEGF and FGF by HUVECs. HUVECs were maintained in culture in MCDB 131 medium containing 10% FBS supplemented with ethanolic extracts of triphala churna for 48 h. The levels of VEGF (**A**) and FGF (**B**) in the medium were estimated by ELISA using anti-VEGF antibody. Values given are the average of five experiments ± SEM. * statistically significant compared to control (*p* < 0.05).

The molecular mechanism of the effect of ethanolic extracts of THL on angiogenesis was analyzed by studying the production of VEGF, which is a key stimulus of angiogenesis, and FGF, another growth factor which plays an important role in regulating angiogenesis. ELISA showed that there was a significant decrease in the amount of VEGF in cells treated with ethanolic extracts of THL compared to control cells. The amount of FGF was significantly decreased in HUVECs treated with THL compared to control. These results are shown in Figure 2.

### 3.5. Effect of Punicalagin on Markers of Angiogenesis

**Figure 3:**
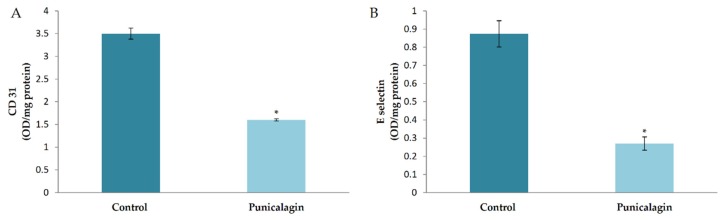
Effect of punicalagin on the production of CD31 and E-selectin by HUVECs. HUVECs were maintained in culture in MCDB 131 medium supplemented with punicalagin (25 µM) for 48 h. The levels of cell-associated CD31 (**A**) and E-selectin (**B**) from the medium were estimated by ELISA using anti-CD-31 and anti-E-selectin, respectively. Values given are the average of five experiments ± SEM. * statistically significant compared to control (*p* < 0.05).

In silico studies showed that the prediction efficacy of punicalagin was high compared to other compounds in THL. To confirm the effect of punicalagin in endothelial cells, the production of CD31 and E-selectin was analyzed in HUVECs treated with punicalagin. Results showed a significant decrease in the amount of CD31 in cells treated with punicalagin compared to controls. Results of the study on the effect of punicalagin on CD31 production by western blot, (given in Supplementary Figure S1) also showed that PA reversed the VEGF-induced upregulation of CD31. E-selectin was down regulated in cells treated with punicalagin in the presence of serum compared to controls (Figure 3).

### 3.6. Effect of Punicalagin on the Production of Angiogenic Growth Factors by HUVECs in Culture

**Figure 4:**
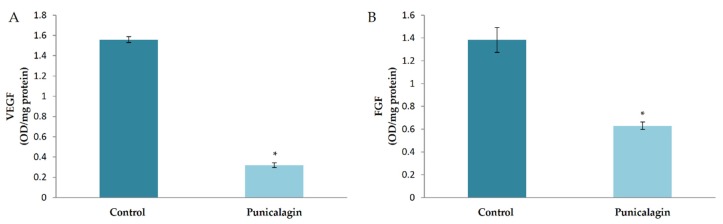
Effect of punicalagin on the production of VEGF and FGF by HUVECs. HUVECs were maintained in culture in MCDB 131 medium containing 10% FBS supplemented with punicalagin (25 µM) for 48 h. The levels of VEGF (**A**) and FGF (**B**) in the medium were estimated by ELISA. Values given are the average of five experiments ± SEM. * statistically significant compared to control (*p* < 0.05).

The effect of punicalagin on the levels of major pro-angiogenic factors was analyzed in HUVECs in culture. The result showed a decrease in the production of VEGF in cells treated with puniclagin compared to control. FGF is another growth factor which plays an important role in regulating angiogenesis. The levels of FGF were also analyzed in HUVECs treated with punicalagin in serum-stimulated conditions by ELISA. The amount of FGF was significantly decreased in HUVECs treated with punicalagin compared to controls, confirming the earlier result (Figure 4).

### 3.7. Effect of Triphala Extract and Punicalagin on Endothelial Cell Migration

**Figure 5:**
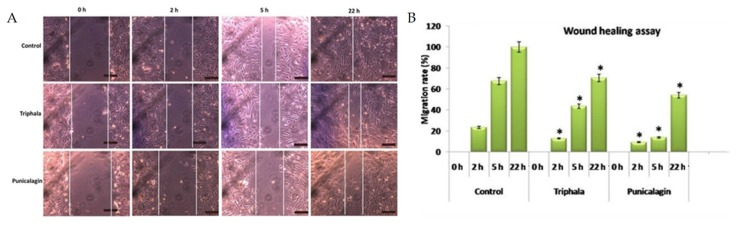
Effect of triphala extract and punicalagin on cell migration. (**A**) Cell migration assay was performed by treating HUVECs with THL (25 µg/mL) and punicalagin (25 µM). Images at different time intervals are shown. (**B**) Width of the wound was measured using Image J Macros software and expressed as migration rate (%). The values given are the average of width of scratch measured at three different areas of each group. * statistically significant compared to control cells (*p* < 0.05).

To study the effect of THL and punicalagin on cell migration, a key event in angiogenesis, a cell migration assay was performed. A scratch was made in the endothelial cell layer and the cells were treated with THL and punicalagin and maintained in culture. Morphological analysis at regular time intervals showed that the migration rate was significantly lower in treated cells when compared to controls (Figure 5). After 22 h of treatment, the wound gap was filled in control cells (100% cell migration), whereas the wound was not completely closed in cells treated with THL (70% cell migration) and punicalagin (54% cell migration). The effect of THL on cell migration was also examined in presence of VEGF. VEGF treatment increased the rate of migration and the gap was filled completely in 6 h, a shorter time than for the untreated control. In cells treated with THL, wound filling took longer; after 21 h the migration rate was only about 70%, suggesting that THL reversed the VEGF-induced upregulation of the rate of cell migration. It appeared that VEGF did not fully rescue the cells from THL-induced retardation of migration (Supplementary Figure S2). The inhibitory effect of THL and punicalagin on endothelial cell migration further indicates their anti-angiogenic property.

### 3.8. Binding of Punicalagin with Crucial Targets

**Figure 6:**
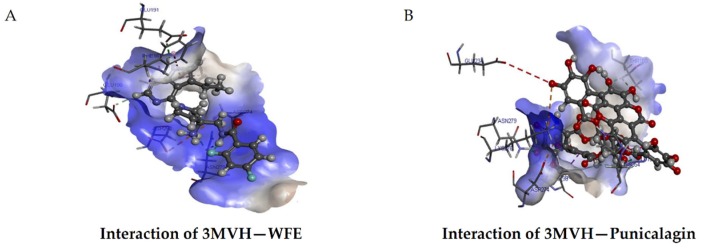
Molecular docking of punicalagin to Akt. Molecular docking of ligands with Akt was done using Discovery studio 4.1. Representative images of the binding of WFE with Akt (**A**) and Punicalagin with Akt (**B**) are shown. Libdock score of Akt–punicalagin: 119.327, number of H bonds: 9.

Punicalagin had more binding affinity than other compounds (Table 1) towards the signaling molecules downstream to VEGF/VEGFR2 signaling involved in angiogenesis. Akt is a key downstream target of VEGF signaling, having more interactions with other signaling molecules. Since punicalagin inhibited angiogenesis in HUVECs, docking of punicalagin with Akt was done using Discovery studio 4.1 to measure the binding affinity of punicalagin to Akt. The docked structures were analyzed to identify the interactions between targets and ligands. Results showed that punicalagin has a high binding affinity with Akt, with a libdock score of 119.3 with nine hydrogen bond interactions. This score was higher than that of the natural ligand (91.9763) in the corresponding PDB entry. Thus, punicalagin, an important constituent of THL, showed the most favorable binding energy against Akt compared to other compounds, and the results are shown in Figure 6.

### 3.9. Effect of Punicalagin on the Levels and Activation of Akt in HUVECs

**Figure 7:**
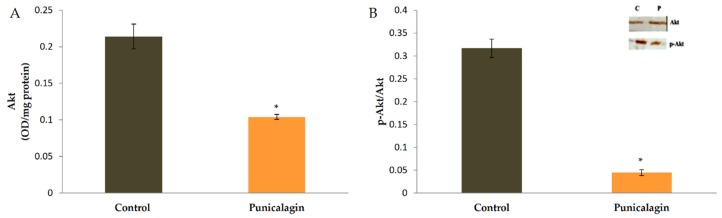
Effect of punicalagin on the production and activation of Akt. HUVECs were maintained in culture in MCDB 131 medium containing 10% FBS supplemented with 25 µM punicalagin for 48 h, as indicated earlier. After treatment, cells were collected and lysed. (**A**) The levels of Akt in cells were determined by ELISA. The values given are the average of five experiments ± SEM. * statistically significant when compared to control (*p* < 0.05). (**B**) The activation of Akt by phosphorylation was analyzed by western blot analysis. Equivalent amount of Akt as determined by ELISA was immunoprecipitated from cell lysate and subjected to immunoblot analysis (inset) for Akt phosphorylation. The intensity of bands was quantitated and plotted as ratio of p-Akt:Akt.

Docking studies revealed that punicalagin can bind to Akt and thus Akt signaling can be blocked. The effect of punicalagin on the level and activation of Akt by phosphorylation was studied by ELISA and western blot analysis. ELISA showed that there was a decrease in the amount of Akt in cells treated with punicalagin in serum-stimulated conditions compared to control cells (Figure 6). Immunoblots were also performed to examine whether there was any change in the activation of Akt by phosphorylation. Equivalent amounts of Akt as determined by ELISA were immunoprecipitated from cell lysate, subjected to western blot analysis, and probed for Akt phosphorylation. In endothelial cells treated with punicalagin, there was a decrease in the level of phosphorylated Akt as compared to controls, suggesting that punicalagin inhibited the activation of Akt in endothelial cells. The results are shown in Figure 7.

## 4. Discussion

Monotarget therapy is less effective in various diseases due to the crosstalk among signaling molecules and the presence of feedback loops among multiple signaling pathways. This issue leads to the suggestion of multi-target therapy. Drug with multi-target action are clinically proven to be more effective than the mono-targeted conventional drugs. The traditional Indian ayurvedic medicinal formulation contains multiple phytocompounds from various medicinal plants that were used for the treatment of several diseases in ancient times. Generally, most of these compounds simultaneously interact with multiple targets in diverse physiological processes, leading to a synergistic multi-targeting effect. This may happen in two ways: either a single constituent of the formulation affects multiple targets at the same time, or multiple components of the formulation affect multiple targets. Considering the first case, the binding affinity of the compound to different targets is very high compared to other constituents. In the second case, binding affinity of the individual compound is different for different targets. In silico docking studies help screen out the binding efficacies of compounds to different targets. In the present study, the approach of predicting the efficacy of a polyherbal combination through in silico docking studies was validated by analyzing the effect of THL on angiogenesis. Docking studies revealed that (a) different small molecular components of the THL dock to different target proteins of the VEGF/VEGFR2 signaling pathway with different binding affinities, and (b) among the 15 principal small molecular components, punicalagin showed highest affinity to 18 targets, chebulagic acid to 7 targets, and isoterchebulin with 2 targets. These molecules showed better docking scores to each target than other phytocompounds of THL, indicating a better binding affinity of each of these molecules to the targets. Based on the docking scores, the possible effect of each of the phytochemicals on various targets in VEGF-induced angiogenesis was predicted by calculating the prediction efficacy. Punicalagin showed highest prediction efficacy, followed by chebulagic acid and isoterchebulin, and the efficacy of THL could be predicted.

VEGF/VEGFR2 signaling activates several downstream signaling pathways, leading to stimulation of multiple events relevant to angiogenesis, such as cell–cell adhesion, cell survival, cell proliferation, and cell migration. VEGF-stimulated PI3K/Akt/NFκB signaling regulates cell survival and cell proliferation by inducing expression of cyclins. Moreover, Akt induces the expression of anti-apoptotic proteins such as Bcl2, XIAP, and surviving, thereby inhibiting caspase-3 and caspase-7. The VEGF/PI3K/Akt/mTOR signaling pathway regulates endothelial cell proliferation, migration, adhesion, and survival [25]. VEGF/VEGFR2 signaling through p38MAPK and focal adhesion kinase (FAK) plays a key role in endothelial cell migration [26]. VEGF/VEGFR2-induced phosphorylation of FAK increases the formation of focal adhesions and is chemotactic for endothelial cells, thereby promoting cell migration [27,28]. Docking studies revealed that THL can target these downstream signaling molecules, as indicated by the following observations. (a) In silico studies showed that the components present in THL showed potential binding affinity with VEGFR2, PI3k, Akt, and mTOR, leading to the inhibition of the VEGF-mediated cell survival pathway. (b) Virtual binding scores indicated that the phytochemicals also possess the ability to bind with Ras, Raf, MEK, and ERK and to deregulate cell proliferation relevant to angiogenesis. (c) Compounds present in THL also target FAK activation and thereby regulate cell migration. The docking of 15 phytochemicals of THL with 27 signaling molecules of the VEGF/VEGFR2 signaling pathway showed that most of the phytochemicals can bind with each of the signaling molecule of VEGF/VEGFR2 pathway. The best interaction between phytochemicals and signaling molecules was analyzed by calculating the prediction efficacies of individual phytochemicals based on docking scores by fixing the threshold docking score at (−)6.00. The highest prediction efficacy was shown by punicalagin, chebulagic acid, isoterchebulin, chebulinic acid, and corilagin compared to other phytochemicals of THL. The prediction efficacy of THL was also examined by deleting low-score alternatives from the screened data. The mode of action of THL on angiogenesis involves the collective effect of multiple phytochemicals, and the prediction efficacy of THL against angiogenic targets was found to be (−)436.7.

Angiogenesis and inflammation are closely related processes capable of potentiating each other [29], and inflammatory mediators regulate angiogenesis. In addition to angiogenesis, inflammation is also a critical component in the development of tumor and its progression, and it can be targeted as a therapeutic strategy for cancer prevention and cancer therapy. During chronic inflammation, chemokines, cytokines, selectins, and growth factors produced by inflammatory cells provide a prerequisite microenvironment favoring tumor growth. In turn, tumor cells regulate immune cell functions such as the production of inflammatory enzymes (COX and LOX), matrix metalloproteases, and chemokines to modulate tumor metastasis [30]. Anti-inflammatory therapy is effective in controlling tumor growth, and anti-inflammatory drugs such as COX-2 inhibitors and 5-LOX inhibitors are widely used to reduce the incidence of cancer and impair tumor growth and metastasis [31].

THL herbal formulation exerts promising anti-inflammatory activity by downregulating production of the inflammatory enzyme COX-2 [32,33]. In addition to this, chebulagic acid, a constituent of THL, has been reported to inhibit COX and 5-LOX [16]. Reports have also shown the anti-inflammatory potential of THL components such as chebulinic acid, ellagic acid, gallic acid, and punicalagin [34,35]. Beta-sitosterol has been reported to exert an anti-inflammatory effect in endothelial cells, and shikimic acid has been shown to inhibit the production of pro-inflammatory cytokines [36,37]. Quinic acid, corilagin, and maslinic acid also possess anti-inflammatory effects in different model systems [38,39,40]. Analysis of the binding affinities of 15 phytochemicals of THL with COX-2 and 5-LOX by docking studies showed that binding affinity was high for punicalagin, chebulagic acid, isoterchebulin, chebulinic acid, and corilagin, suggesting that these phytochemicals contribute to both the anti-angiogenic and anti-inflammatory activities of THL.

The ligands chebulagic acid, chebulinic acid, punicalagin, and isoterchebulin showed better docking scores, indicating higher binding affinity. Other ligands did dock to different targets, but with poor docking scores, indicating low binding affinity. When these are present along with other ligands with higher binding affinity, as in THL formulation, the binding of these low-affinity ligands to the targets in VEGF/VEGFR2 pathway may be low. This sort of differential effect of the components of polyherbal formulations can be predicted by the in silico approach employed here.

Angiogenesis is regulated by the crosstalk between the VEGF/VEGFR2 signaling pathway and multiple signaling pathways. Akt and β-catenin are the most important signaling molecules contributing to the convergence of multiple signaling pathways regulating angiogenesis. This has been suggested to be one of the reasons for the development of resistance to anti-angiogenic therapy. Docking studies have shown that the key components in these convergent points are targets of phytocompounds present in THL. The THL component punicalagin showed binding affinity to AkT and chebulagic acid to β-catenin.

Validation of the in silico prediction studies by in vitro cell-based assays further confirmed the inhibitory effect of THL on angiogenesis. In vitro studies showed that THL inhibits angiogenesis, as evidenced by (a) decreased production of CD31 and E-selectin in endothelial cells treated with THL, (b) inhibition of cell migration as indicated by wound healing assay, and (c) downregulation of VEGF and FGF in endothelial cells. CD31 and E-selectin are cell adhesion molecules that mediate endothelial cell–cell contact during angiogenesis [41,42]. Decreased levels of CD31 and E-selectin on treatment with THL may lead to inhibition of cell–cell contact and the endothelial tube formation relevant to angiogenesis. Endothelial cell migration is a critical event in angiogenesis as it is necessary for the formation of tubular structures, and thereby formation of new blood vessels [43]. The inhibition of endothelial cell migration by THL further confirmed the anti-angiogenic effect of THL. VEGF and FGF are key regulators of neovascularisation [44]. Results showed that THL mediates its anti-angiogenic effect by downregulating VEGF and FGF. Reversal of VEGF-induced upregulation of CD31 and cell migration by THL further suggest that THL affects VEGF-mediated angiogenesis.

The prediction efficacy results indicated that components of THL, particularly punicalagin and chebulagic acid, contribute to the anti-angiogenic effect of the THL formulation. Chebulagic acid and punicalagin target and interact with components of signaling pathways downstream to VEGF/VEGFR2 signaling, such as PI3K/Akt, FAK/paxillin, and p38MAPK/ERK, and thereby inhibits endothelial cell proliferation, cell adhesion, and cell migration, respectively, during angiogenesis. Downregulation of angiogenic markers CD31 and E-selectin and angiogenic growth factors VEGF and FGF, and inhibition of migration of endothelial cells on treatment with punicalagin further indicated its inhibitory effect on angiogenesis. Further studies to identify the molecular mechanism underlying the angiostatic effect of punicalagin indicated that it modulates the PI3K/Akt signaling pathway. Docking analysis was validated by in vitro studies, which showed that the activation of Akt by phosphorylation was significantly decreased in cells treated with punicalagin, confirming the involvement of Akt in mediating the anti-angiogenic effect of punicalagin. The anti-angiogenic effect of chebulagic acid has been already reported by our laboratory [15,17]. Chebulagic acid inhibits angiogenesis by downregulating VEGF, VEGFR2, GSK3β, VE-cadherin, and the VE-cadherin–β catenin signaling necessary for the stabilization of endothelial cell–cell adhesion during angiogenesis.

The present study provides a new approach to predict the synergism of active components of a polyherbal formulation and the efficacy of the same, based on computational approaches using parameters such as docking score and prediction efficacy. This approach could be useful for exploring the concurrent activities of multiple phytochemical components of a polyherbal formulation and also to find the component with the best therapeutic potential. Application of this approach in the area of drug discovery will contribute to advances in therapeutic strategies based on natural products.

Anti-VEGF therapy is used for blocking angiogenesis in tumor tissues, but resistance to anti-VEGF therapy has been reported [45]. This might be due to the occurrence of VEGF-independent signaling pathways which converge with the downstream components of VEGF/VEGFR2 signaling pathway such as Akt, as described in the VEGF/VEGFR2 signaling pathway map [9]. Since THL, a polyherbal formulation, inhibits angiogenesis by blocking multiple components of VEGF/VEGFR2 signaling pathway, it can be used as an adjuvant along with anti-VEGF therapy as an alternative therapeutic strategy in anti-VEGF-resistant conditions.

## 5. Conclusions

The efficacy of the multiple phytochemicals of THL in targeting multiple signaling molecules of VEGF/VEGFR2 signaling pathway and the cumulative efficacy of the THL formulation against angiogenic targets were examined in the present study using in silico and in vitro methods. The molecular docking studies revealed the prediction efficacy of THL and its constituent phytochemicals against multiple targets involved in angiogenesis, indicating the interaction of various phytochemicals of THL with multiple angiogenic regulators. Validation of docking results using angiogenesis assays confirmed the inhibitory effect of THL on angiogenesis. THL inhibits angiogenesis by blocking various signaling proteins involved in the VEGF pathway, and this is mediated by the synergistic action of multiple phytochemicals present in the polyherbal formulation. The current work proposed a new approach to determine the potential efficacy of polyherbal formulations against various pathological conditions.

## Figures and Tables

**Table 1:** Binding affinities of 15 ligands with 27 targets.

	L1	L2	L3	L4	L5	L6	L7	L8	L9	L10	L11	L12	L13	L14	L15
**T1**	−14.3	−12.1	−6	−16.1	−14.7	−7.1	−7.4	−6.7	−5.9	−5.6	−6	−5	−8.6	−7.6	−7.7
**T2**	−13.6	−11.6	−5.7	−14.4	−13.1	−6.5	−6.9	−6.9	−5.5	−5.5	−5.6	−4.2	−7.8	−7.4	−8.1
**T3**	−15.4	−15.1	−5	−15.7	−15.3	−8	−7.8	−6	−4.8	−5	−5	−5.3	−7.9	−8.2	−7.8
**T4**	−17.9	−15.2	−6.2	−17.4	−16.6	−7.5	−8.4	−6.7	−5.7	−5.7	−5.6	−5	−8.7	−7.7	−7.4
**T5**	−14.6	−12.9	−5.6	−15	−14.1	−7.3	−7.2	−6.9	−5.2	−5.2	−5.7	−4.2	−9	−8.5	−7.4
**T6**	−13	−12.6	−7.1	−15.3	−14.5	−6	−10.6	−6.8	−7.1	−7.2	−7.6	−6.2	−8.1	−8	−7.5
**T7**	−13	−12	−5.4	−13.7	−13.8	−6.1	−6.7	−6.1	−4.8	−5	−5.1	−4	−7.9	−6.4	−6.2
**T8**	−20.4	−14.6	−6.2	−15.1	−14.1	−8.6	−8.7	−8.2	−6.7	−6.7	−6.4	−6.8	−7.6	−8.9	−8
**T9**	−14.5	−14.7	−6.2	−16.2	−16	−7.1	−8.7	−7.7	−5.8	−5.8	−5.8	−4.8	−9.9	−8.3	−7.8
**T10**	−14	−12.7	−6.5	−14.4	−13.1	−7.7	−7.9	−6.9	−6.5	−6.5	−6.9	−5.5	−8.5	−7.9	−7.5
**T11**	−16.6	−14.4	−5.9	−17.9	−16.8	−7.7	−7.3	−6.4	−5.3	−5.8	−5.7	−4.7	−8.7	−9.4	−8.8
**T12**	−13.7	−12.2	−5.7	−15.8	−14.9	−7.2	−9.2	−6.7	−5.2	−5.9	−5.3	−5.3	−8.7	−8.3	−7.9
**T13**	−14.3	−12.7	−5.8	−15	−14.2	−6.9	−7.7	−6.6	−5.5	−5.9	−5.8	−6.9	−8.2	−8.1	−7.7
**T14**	−15.3	−13.2	−5.6	−15	−14.2	−8	−8.4	−7.4	−5.6	−5.6	−5.9	−6.6	−8.1	−9.1	−8.2
**T15**	−16.5	−12.4	−5.9	−20	−17.8	−7.5	−9.2	−6.6	−5.6	−6.1	−5.4	−4.6	−7.3	−8.5	−8.5
**T16**	−14.9	−12.7	−5.5	−15.2	−13.6	−6.5	−8.1	−7.4	−5.4	−5.1	−5.7	−4.3	−8.8	−7.9	−7.9
**T17**	−18.4	−15.2	−6.3	−17.2	−16.1	−8.7	−8.8	−8.5	6.4	−6.3	−6.6	−5.7	−10	−9.2	−9.4
**T18**	−12.7	−10.8	−5	−13.3	−12.5	−6.7	−7	−6	−4.8	−4.8	−5.4	−3.4	−7.1	−7.2	−7.3
**T19**	−17.8	−15.3	−6.2	−17.9	−17.3	−8	−8.3	−7.3	−6	−6.1	−6.5	−5.1	−9.9	−9.4	−9.2
**T20**	−18	−16.9	−7.1	−19.5	−17.3	−9.4	−8.8	−7.6	−6.6	−7	−7	−6.3	−9.7	−9.1	−9
**T21**	−19.1	−13.9	−6.4	−16.8	−15.7	−8.8	−10.3	−7.8	−6.2	−6.7	−6.1	−6.2	−8.7	−9.1	−8.8
**T22**	−12.1	−11.1	−5.1	−12.6	−12.3	−6.2	−7	−5.5	4.6	−5.2	−4.5	−5.2	−6.9	−7	−6.9
**T23**	−13.4	−12.3	−4.9	−14.2	−13.7	−6.4	−7	−5.9	−5	−4.7	−4.9	−3.8	−7.9	−7.8	−7.4
**T24**	−17.1	−14.9	−6.9	−18.4	−18.4	−8.8	−8.2	−7	−7	−7.1	−7.4	−5.9	−9.4	−9.5	−8.7
**T25**	−14.4	−12	−5.6	−12.2	−11.1	−5.1	−6.1	−5.6	−4.9	−5	−4.8	−3.9	−6.6	−6.1	−5.9
**T26**	−16.5	−13.7	−7.2	−17.1	−16.5	−7.9	−9	−7.7	−6.9	−6.9	−7.1	−5.5	−8.7	−8.5	−8.3
**T27**	−13.9	−13.1	−5.4	−15.7	−15.3	−7.1	−7	−6.4	−5	−5.2	−5	−4.6	−8.6	−8.2	−8.2

L1–L15 indicate chebulagic acid, chebulinic acid, gallic acid, punicalagin, isoterchebulin, betasitosterol, ellagic acid, chebulic acid, shikimic acid, dehydroshikimic, quinic acid, triacontanoic acid, corilagin, maslinic, and arjunolic acid respectively. T1–T27 represent the targets in the following order: VEGF A (1VPF), VEGF R1 (1RV6), VEGFR2 (1Y6A), NRP 1(2QQM), NRP 2(2QQO), PKCγ (2UZP), RAS (5P21), RAF 1(3OMV), MEK (3EQC), ERK (2Y9QP), PLCγ (4EY0), FAK (4I4E), Cdc42 (2KBO), p38MAPK (1W82), MAPKAPK (3FHR), SRC (1A1C), PI3K (3ZIM), RAC (1MH1), AKT/PKB (2X18), eNOS (1M9M), paxillin (2VZI), Hsp27 (3Q9P), axin (1EMU), GSK 3β (1Q3W), β-catenin (1LUJ), MMP9 (1L6J), and MMP3 (1UEA). The PDB id of each target is provided in parentheses.

**Table 2:** Prediction efficacy of triphala churna (THL) against angiogenic targets.

SI. No.	Phytocompound	PubChem ID	Prediction Efficacy (kcal/mol)
1.	Punicalagin	44584733	−424.8
2.	Chebulagic acid	442674	−414.3
3.	Isoterchebulin	16143735	−400.9
4.	Chebulinic acid	72284	−360.1
5.	Corilagin	73568	−226.3
6.	Ellagic acid	5281855	−217.1
7.	Maslinic acid	73659	−207.3
8.	Arjunolic acid	73641	−206.4
9.	Beta sitosterol	222284	−192.7
10.	Chebulic acid	12302892	−167.7
11.	Gallic acid	370	−72.3
12.	Dehydroshikimic acid	5460360	−66.6
13.	Quinic acid	6508	−61.6
14.	Shikimic acid	8742	−59.4
15.	Triacontanoic acid	10471	−39
**Anti-Angiogenic Prediction Efficacy of Triphala**			−436.7

The prediction efficacy (PE) of a phytocompound was calculated by summing up its binding affinity values with all the targets obtained by docking with Autodock Vina. The number of compounds with high docking scores for each target was taken to calculate the PE of the polyherbal formulation.

**Table 3:** Binding affinity of phytocompounds of THL against mediators of inflammation.

	L1	L2	L3	L4	L5	L6	L7	L8	L9	L10	L11	L12	L13	L14	L15
**T1**	16.1	13.7	6.1	17.1	15.8	6.7	23.8	6.9	6	6.1	6	4.2	9.5	8.9	7.9
**T2**	14.6	14.3	6.4	16.7	16.2	7.9	9	7	6.5	6.3	6.1	6.7	9.2	8.4	8.1

Negative binding affinities of 15 ligands with targets. Here, L1–L15 indicate chebulagic acid, chebulinic acid, gallic acid, punicalagin, isoterchebulin, betasitosterol, ellagic acid, chebulic acid, shikimic acid, dehydroshikimic, quinic acid, triacontanoic acid, corilagin, maslinic, and arjunolic acid, respectively. Target T1 represents COX-2 (3LN1) and T2 represents 5-LOX (3O8Y).

**Table 4:** Prediction efficacies of phytocompounds and triphala churna on inflammation.

SI. No.	Phytocompound	PubChem ID	Prediction Efficacy (kcal/mol)
1.	Punicalagin	44584733	−33.8
2.	Isoterchebulin	16143735	−32
3.	Chebulagic acid	442674	−30.7
4.	Chebulinic acid	72284	−28
5.	Corilagin	73568	−18.7
6.	Maslinic acid	73659	−17.3
7.	Ellagic acid	5281855	−17
8.	Arjunolic acid	73641	−16
9.	Beta sitosterol	222284	−14.6
10.	Chebulic acid	12302892	−13.9
11.	Gallic acid	370	−12.5
12.	Shikimic acid	8742	−12.5
13.	Dehydroshikimic acid	5460360	−12.4
14.	Quinic acid	6508	−12.1
15.	Triacontanoic acid	10471	−6.7
**Anti-Inflammation Prediction Efficacy of Triphala**			**−33.8**

The prediction efficacy (PE) of a phytochemical compound was calculated by summing up its binding affinity values with all the targets obtained by docking with Autodock Vina. The number of compounds with high docking scores for each target was taken to calculate the PE of the ayurvedic formulation.

## Supplementary Materials

The following are available online at https://www.mdpi.com/2218-273X/10/2/177/s1, Figure S1: Effect of ethanolic extract of Triphala and punicalagin on angiogenesis during VEGF stimulated conditions, Figure S2: Effect of THL on endothelial cell migration in presence of VEGF.
