# Supplementary material for: Multiple Targets Directed Multiple Ligands: An In Silico and In Vitro Approach to Evaluating the Effect of Triphala on Angiogenesis
Source: Biomolecules. 2020 Jan 23;10(2):177. doi: 10.3390/biom10020177 (PMC7072423; doi:10.3390/biom10020177)
Supplement: Supplementary file 1 [file biomolecules-10-00177-s001.pdf]

Supplementary data

Effect of ethanolic extract of Triphala and punicalagin on angiogenesis during VEGF stimulated conditions: Changes in CD31 in HUVECs

In order to study the effect of Triphala and punicalagin on angiogenesis during VEGF stimulated conditions, western blot analysis of CD 31 – a major angiogenic marker was examined. Endothelial cells were treated with VEGF, VEGF +THL and VEGF + PA and maintained in culture. Normal cells served as control. After 48 hours, cells were harvested and western blot of CD 31 was performed. Our result showed that there was an increase in the level of CD31 in cells treated with VEGF compared to control cells. Further, a decrease in the production of CD 31 was seen in cells treated with THL and PA compared to that treated with VEGF alone. Among them, cells treated with PA showed maximum decrease in the production of CD 31. In cells treated with VEGF and THL, the CD 31 levels were rescued to near normal levels. These results suggest that THL and punicalagin could reverse the VEGF-induced up-regulation of CD31.

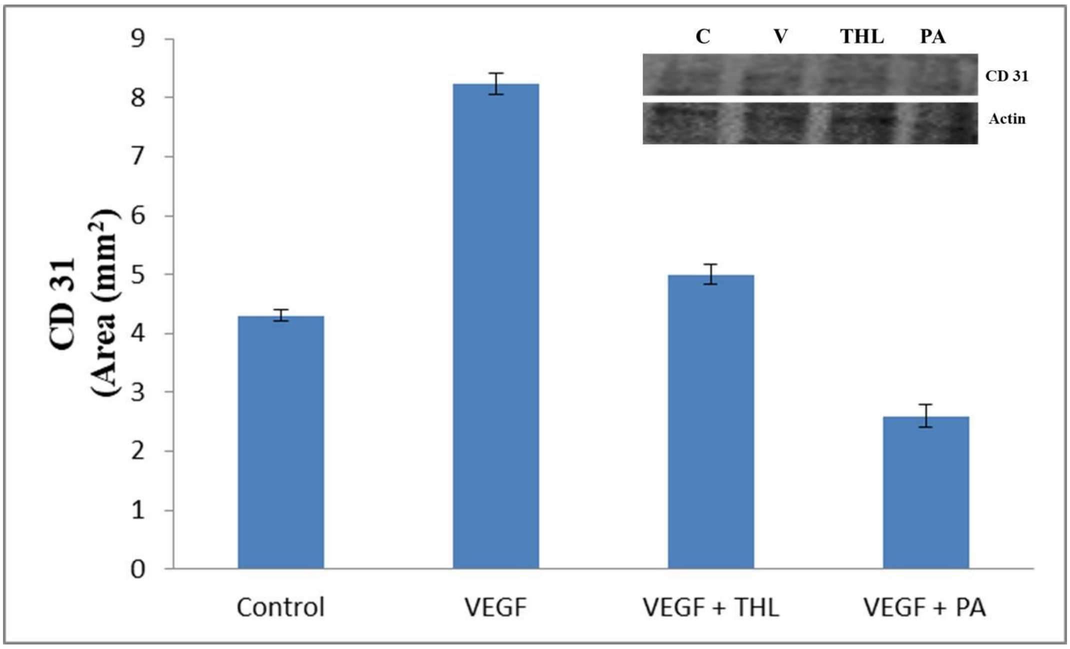

Figure S1: Effect of ethanolic extract of Triphala and punicalagin on angiogenesis during VEGF stimulated conditions. Endothelial cells were isolated and treated with VEGF, VEGF +THL and VEGF + PA and maintained in culture. Normal cells served as control. After 48 hours, cells were harvested and western blot of CD 31 was performed. The bands were quantitated using image lab software and expressed as area (mm^2). Values given are mean of 3 experiments ± SEM.

Effect of Triphala extract on endothelial cell migration in presence of VEGF

To study the effect of triphala on endothelial cell migration in the presence of VEGF, scratch assay/cell migration assay was performed. A scratch was made in the endothelial cell layer and the cells were treated with VEGF along with triphala extract(THL) .Morphological analysis was performed at regular intervals and it showed that cell migration was decreased in cells treated with THL in presence of VEGF compared to cells treated with VEGF alone. Wound gap was filled in cells treated with VEGF within 6 hours of treatment whereas the gap was not filled completely in cells treated with VEGF+THL even during 21 hours of treatment. Inhibitory effect of THL on endothelial cell migration indicated the anti angiogenic property of these compounds. These results suggested that decreased cell migration by THL (mentioned in the manuscript) was not fully rescued by the presence of VEGF.

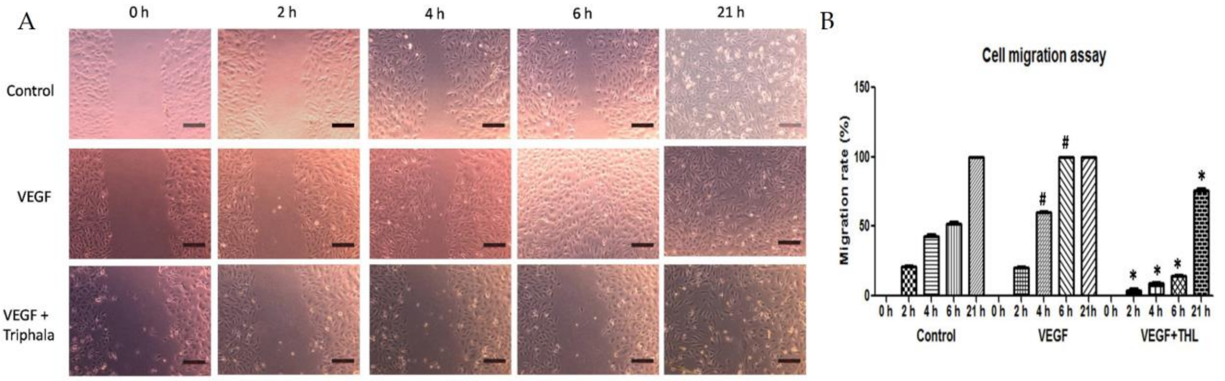

Figure S2: Effect of THL on endothelial cell migration in presence of VEGF. Endothelial cells were maintained in culture in EGM medium supplemented with 10% FBS. (A) Cell migration assay was performed by treating endothelial cells with THL (25µg/mL) in presence of VEGF (20ng). Images at different time intervals were given. (B) Width of the wound was measured using Image J Macros software and expressed as migration rate (%). The values given are the average of width of scratch measured. *statistically significant compared to cells treated with VEGF ($p<0.05$).
